# Clinical outcomes and quality of life associated with eculizumab in patients with late-onset myasthenia gravis

**DOI:** 10.3389/fimmu.2026.1834680

**Published:** 2026-06-12

**Authors:** Li Wang, Meijie Qu, Xi Rong, Xupeng Sun, Yunbin Zhao, Shuzhou Huang, Min Liu

**Affiliations:** 1 Department of Neurology, Affiliated Hospital of Qingdao University, Qingdao, China; 2 Department of Orthopedics, Qingdao University Affiliated Yantai Yuhuangding Hospital, Yantai, China

**Keywords:** acetylcholine receptor antibody, eculizumab, late-onset myasthenia gravis, refractoriness, therapy, quality of life

## Abstract

**Introduction:**

Late-onset myasthenia gravis (LOMG) features prominent immunosenescence, high myasthenic crisis risk, and refractoriness due to comorbidities limiting conventional immunosuppressants, leading to poor prognosis. Eculizumab, which blocks complement C5 activation, may provide rapid symptom improvement and has shown an acceptable safety profile in previous studies, holding potential for LOMG management. Although the REGAIN trial and some real-world studies have demonstrated the efficacy of eculizumab, evidence for its use in patients with refractory LOMG is scarce. This study aimed to evaluate the clinical outcomes, safety profile, and impact on quality of life of eculizumab treatment in patients with refractory acetylcholine receptor antibody-positive [AChR-Ab(+)] generalized LOMG.

**Methods:**

This study retrospectively enrolled 31 patients with refractory AChR-Ab(+) generalized LOMG treated with eculizumab for ≥2 months. Primary outcome was change in myasthenia gravis activities of daily living (MG-ADL) score analyzed using mixed models for repeated measures. Secondary outcomes included 15-item Revised Myasthenia Gravis Quality of Life Questionnaire (MG-QOL 15r) score, Minimal Symptom Expression (MSE) response rate, clinically meaningful improvement (a decrease of ≥3 points in MG-ADL, CMI) in ADL, and concomitant immunosuppressant dosage reduction. Safety was assessed via drug-related adverse events.

**Results:**

The mean age of onset in the 31 patients with refractory LOMG was 62.87 ± 5.95 years, with a mean follow-up duration of 10 ± 4.29 months after eculizumab treatment. Compared with baseline, the MG-ADL score was reduced by 2.976 points at Month 1 and 5.430 points at Month 12 (both P<0.001); MG-QOL 15r scores reduced by 7.724 and 15.343 points respectively (both P<0.001). Alluvial plots showed that MG-ADL and MG-QOL 15r scores continuously shifted to lower ranges with treatment. MSE cumulative rate was 22.58% at Month 1 and 72.24% at Month 12, with CMI rate rose from 51.61% to 96.41%. Mean prednisone daily dose decreased by 7.574 mg/day (P<0.001). Sensitivity analyses using multiple imputation and conservative baseline observation carried forward (BOCF) yielded consistent results, supporting the robustness of the findings. One patient reported headache (CTCAE 1 grade), no serious adverse events occurred.

**Conclusion:**

Eculizumab was associated with clinical improvement in patients with refractory AChR-Ab(+)-generalized LOMG. In this cohort, patients demonstrated rapid symptom reduction and improvements in long-term quality of life.

## 1 Introduction

Myasthenia gravis (MG) is an acquired autoimmune disorder characterized by autoantibody-mediated impairment of signal transmission at the postsynaptic membrane of the neuromuscular junction (1). Approximately 80–85% of patients with MG are positive for acetylcholine receptor antibodies (AChR-Ab), while the remaining cases include those positive for MuSK antibodies, LRP4 antibodies, or antibody-negative patients (2, 3). MG is classified into early-onset myasthenia gravis (EOMG) and late-onset myasthenia gravis (LOMG, defined as onset at ≥ 50 years of age) (4). The incidence of LOMG has steadily increased in recent years (5). LOMG is associated with distinct immunosenescence features, high risk of infection, and myasthenic crises (5, 6). Furthermore, the widespread presence of multiple comorbidities, such as hypertension and diabetes, severely restricts the use of glucocorticoids and conventional immunosuppressants (e.g., tacrolimus and mycophenolate mofetil), leading to a refractory state characterized by an inadequate response to conventional immunosuppressive therapies, intolerance to adverse effects, or dependence on high-dose glucocorticoids (5–8). Refractory LOMG is often accompanied by symptom fluctuations, recurrent myasthenic crises, and superimposed treatment-related side effects, which result in significant impairment of health-related quality of life (HRQOL) (9, 10). Therefore, a novel therapeutic strategy with a rapid onset of action and favorable safety is urgently needed.

The LOMG population is predominantly AChR antibody-positive (6), and these antibodies mainly belong to the IgG1 and IgG3 subclasses, which possess strong complement-activating capacity (11). Its pathogenic mechanism involves not only the functional blockade of acetylcholine receptors but also the activation of complement C5 through the complement cascade reaction, which forms membrane attack complexes (MAC) and causes structural damage to the neuromuscular junction (NMJ) (12). Persistent complement C5 activation is a key mechanism underlying the chronic refractory course of this condition. Complement inhibitors effectively block this pathway, alleviate symptoms, and control disease progression (13). Eculizumab is a recombinant humanized monoclonal antibody that targets the terminal complement C5 (14–16). It specifically blocks the cleavage of C5 and inhibits the formation of MAC and the subsequent damage to the NMJ, thereby achieving rapid symptom relief (14, 15, 17). Eculizumab was approved in China in June 2023 for the treatment of refractory generalized myasthenia gravis (gMG) that is positive for AChR-Ab. The REGAIN trial and its open-label extension study confirmed that eculizumab has favorable efficacy and safety in refractory AChR-Ab-positive MG (18, 19). Compared to conventional immunosuppressive regimens, which have limited efficacy, heavy side effects, and a substantial burden on the quality of life, eculizumab has a rapid onset of action and is less likely to induce extensive immunosuppression. Thus, it may represent a potential therapeutic alternative for patients with refractory LOMG, impaired immune function, and poor tolerance to conventional therapy, though further evidence is needed to establish its comparative efficacy.

Based on real-world data, this study aimed to investigate the clinical outcomes, safety profile, and the quality of life improvements associated with eculizumab in patients with refractory generalized LOMG, and to provide an observational reference for the precise treatment of geriatric myasthenia gravis.

## 2 Methods

### 2.1 Study design

This single-center, retrospective, observational study was approved by the Ethics Committee of the Affiliated Hospital of Qingdao University. Between November 2023 and January 2026, 38 patients with refractory AChR-Ab-positive generalized LOMG received eculizumab treatment. Of these, 31 patients who continued treatment for at least 2 months were included in the primary analyses of clinical outcomes, quality of life, and corticosteroid dose. All 38 treated patients were included in the safety analysis. Follow-up visits were conducted at baseline and Month 1, 3, 6, and 12 after treatment initiation, and relevant data were collected. The primary endpoint of this study was the change in the myasthenia gravis activities of daily living (MG-ADL) score from baseline to each follow-up timepoint. The secondary endpoints included the change in the 15-item Revised Myasthenia Gravis Quality of Life Questionnaire (MG-QOL 15r) score from baseline, proportion of patients achieving Minimal Symptom Expression (MSE), dosage reduction of conventional immunosuppressants such as glucocorticoids, and safety of eculizumab. A reduction of ≥3 points in the MG-ADL score from baseline was defined as clinically meaningful improvement (CMI). Minimal symptom expression (MSE) was defined as an MG-ADL score of 0 or 1.

### 2.2 Study subjects

All 38 patients met the diagnostic criteria for AChR-Ab(+)-generalized refractory LOMG at baseline. The inclusion criteria were as follows: (1) age of disease onset ≥50 years (LOMG); (2) clinically diagnosed generalized MG; (3) positive for AChR-Ab; (4) met the criteria for refractory MG. According to the international guideline, patients were considered to have refractory MG if they met any of the following criteria: a) inadequate therapeutic response after receiving adequate and full-course conventional immunosuppressive therapy, b) inability to continue conventional immunotherapy due to adverse drug reactions, c) repeated dependence on intensive therapies, such as intravenous immunoglobulin (IVIG) or plasma exchange (PE), and d) occurrence of myasthenic crisis during the disease course. In this study, insufficient response to adequate conventional immunotherapy was defined as inadequate clinical response after the following treatment exposure: prednisone 0.3–0.5 mg/kg/day for at least 12 weeks; tacrolimus 2–3 mg/day for at least 12 weeks, with at least one trough concentration exceeding 4.8 ng/mL; or mycophenolate mofetil 0.5–1.0 g twice daily for at least 24 weeks. Relapse during corticosteroid tapering after adequate full-course immunotherapy also falls under this criterion. The exclusion criteria were as follows: use of IVIG within 2 months before the initiation of eculizumab treatment, use of B-cell-depleting agents or other biological agents within 6 months, presence of active infection, or a history of allergy to biological agents. Baseline demographic and clinical data of the patients were collected, including age at onset, sex, disease duration, comorbidities, MG-ADL score, MG-QOL 15r score, Myasthenia Gravis Foundation of America (MGFA) classification, and the use of pyridostigmine bromide, glucocorticoids, and non-steroidal immunosuppressants (NSISTs).

### 2.3 Treatment regimen

Eculizumab treatment consisted of an induction phase (four weeks) and a maintenance phase. During the induction phase, the patients received an intravenous infusion of 900 mg eculizumab once weekly for four consecutive weeks. After the induction phase, the patients entered the maintenance phase, in which a single intravenous infusion of 1200 mg eculizumab was administered at week 5, followed by 1200 mg eculizumab intravenously every two weeks thereafter. All patients who received eculizumab treatment were vaccinated with the ACYW135 meningococcal polysaccharide vaccine two weeks before the initiation of eculizumab treatment or received prophylactic antibiotics during treatment.

Patients continued to take pyridostigmine bromide, glucocorticoids, and non-steroidal immunosuppressants as they did at baseline, and the dosages were adjusted or the drugs were discontinued according to changes in the patient’s condition, with occasional modifications necessitated by institutional drug availability.

### 2.4 Safety assessment

Adverse events (AEs) were graded according to the Common Terminology Criteria for Adverse Events (CTCAE) version 5.0. Safety data were collected retrospectively from medical records, including documentation from scheduled outpatient visits, hospitalization records, laboratory results, and patient-reported symptoms. While no predefined systematic infection surveillance protocol (such as those typically used in prospective clinical trials) was implemented in this study, infection events were identified based on clinically documented diagnoses recorded in the medical charts.

### 2.5 Statistical analysis

Descriptive statistical analysis was used for baseline variables in this study. Categorical variables were presented as frequencies and percentages, while continuous variables were described as mean ± standard deviation (SD), median, and interquartile range (IQR).

Longitudinal changes in MG-ADL, MG-QOL 15r, and corticosteroid dose were analyzed using mixed models for repeated measures (MMRM), with visit as a categorical fixed effect and baseline as the reference. A compound symmetry covariance structure was used. Least Significant Difference(LSD) test was used for pairwise comparisons. For MG-ADL and MG-QOL 15r, models were adjusted for age, baseline MGFA classification, time-varying corticosteroid dose, conventional immunosuppressant use, and pyridostigmine dose. For corticosteroid dose, models were adjusted for age, baseline MGFA classification, conventional immunosuppressant use, and pyridostigmine dose. In the primary analysis (n=31), all available observations from patients who remained on eculizumab treatment for at least 2 months were included under the missing-at-random assumption. Two sensitivity analyses were performed. First, to assess the influence of missing data on the primary findings, a multiple imputation sensitivity analysis (n=31) was performed with 50 imputed datasets, followed by the same MMRM models and pooling of estimates. Second, to address potential selection bias related to early discontinuation, a conservative baseline observation carried forward (BOCF) sensitivity analysis (n=38) was performed in the all-treated population, including all 38 patients who received eculizumab. For the seven patients who discontinued eculizumab before 2 months, all post-baseline MG-ADL, MG-QOL 15r, and corticosteroid dose values at Month 1, 3, 6, and 12 were imputed using their respective baseline values, assuming no clinical improvement or steroid-sparing effect. The same MMRM specification was then refitted. The results are presented as estimated marginal means (EMMs) and 95% confidence intervals (95% CI).

Time-to-event analysis was performed using the Kaplan-Meier method to plot the cumulative response rate curve for MSE achievement after initiating eculizumab treatment, and patients lost to follow-up were censored in the time-to-event analyses.

A two-tailed α level of 0.05 was set as the threshold for statistical significance. All statistical analyses were performed using SPSS Statistics 29.0.2.0 and Prism 10.1.2.

## 3 Results

### 3.1 Study population

Among 1108 patients with MG treated at the Affiliated Hospital of Qingdao University from November 2023 to January 2026, 581 (52.44%) were LOMG patients with an age of onset of ≥50 years, of whom 38 received eculizumab treatment, among them, seven patients with a treatment duration of less than two months.

The reasons for treatment discontinuation in the seven patients with a treatment duration of less than two months were as follows: one patient voluntarily discontinued the drug after symptom improvement to MSE, three patients discontinued treatment for financial reasons, two patients switched to efgartigimod due to poor therapeutic efficacy, and one patient had incomplete data due to lost medical records.

The distribution of patients meeting each refractory MG criterion is summarized in **Table 1**.

**Table 1:** Distribution of patients meeting specific criteria for refractory myasthenia gravis.

Classification of refractory MG criteria	All treated patients (n=38)	Primary analysis cohort (n=31)	Early discontinuation cohort (n=7)
(a)inadequate therapeutic response after receiving adequate and full-course conventional immunosuppressive therapy.	22(57.89%)	18(58.06%)	4(57.14%)
(b)inability to continue conventional immunotherapy due to adverse drug reactions.	29(76.31%)	24(77.41%)	5(71.42%)
(c)repeated dependence on intensive therapies, such as IVIG or PE.	14(36.84%)	11(35.48%)	3(42.85%)
(d) occurrence of myasthenic crisis during the disease course.	5(13.15%)	4(12.9%)	1(14.28%)
Total enrolled refractory MG patients	38(100%)	31(100%)	7(100%)
Number of patients meeting two or more criteria	22(57.89%)	18(58.06%)	4(57.14%)

Data are presented as n (%). The primary analysis cohort included patients who continued eculizumab for at least 2 months. The early discontinuation cohort included patients who discontinued eculizumab before 2 months.

### 3.2 Baseline characteristics

Baseline characteristics of all 38 treated patients, stratified by primary analysis cohort and early discontinuation cohort, are presented in **Table 2**. In the all-treated cohort, the mean age at disease onset was 63.97 ± 7.06 years, and 19 patients (50%) were male. The median disease duration was 27 months (IQR, 10–60). Regarding MGFA classification, 12 patients (31.57%) were classified as class II, seven (18.42%) as class III, and 19 (50%) as class IV. The median baseline MG-ADL and MG-QOL 15r scores were 7.5 (IQR, 5–10) and 22 (IQR, 16–30), respectively. In the main analysis cohort, the mean age of onset was 62.87 ± 5.95 years, and 45.16% of the patients had an age of onset of ≥65 years. The median disease duration was 27 (IQR 9–60) months, and there were slightly more female patients (51.61%). All patients had comorbidities, with hypertension being the most common (51.61%), followed by diabetes (38.71%), thymoma (29.03%), sleep disorders (29.03%), other malignant tumors (22.58%), thyroid diseases (19.35%), and peptic ulcers (19.35%). The median baseline MG-ADL and MG-QOL 15r scores were 8 (IQR 5–11) and 22 (IQR 16–31), respectively. The most common MGFA classification was type IV (54.84%), followed by types II (32.26%) and III (12.9%). In terms of concomitant conventional immunotherapies (cholinesterase inhibitors, glucocorticoids, and non-steroidal immunosuppressants), five patients were treated with glucocorticoids alone at baseline, 12 patients received a combination of glucocorticoids and tacrolimus, four patients received a combination of glucocorticoids and mycophenolate mofetil, and five patients received a combination of tacrolimus and mycophenolate mofetil. All 31 patients were administered pyridostigmine bromide.

**Table 2:** Baseline characteristics of all eculizumab-treated patients.

Characteristic	All treated patients	Primary analysis cohort	Early discon-tinuation cohort
Number of patients, n	38	31	7
Sex, n (%)			
Male	19(50%)	15(48.39%)	4(57.14%)
Female	19(50%)	16(51.61%)	3(42.85%)
Age at disease onset, years, mean ± SD	63.97 ± 7.06	62.87 ± 5.95	68.85 ± 9.85
50-64,n (%)	19(50%)	17(54.84%)	2(28.57%)
≥65,n (%)	19(50%)	14(45.16%)	5(71.42%)
Disease duration, months, median (IQR)	27(10,60)	27(9,60)	14(13,72)
MGFA classification, n (%)			
II	12(31.57%)	10(32.26%)	2(28.57%)
III	7(18.42%)	4(12.90%)	3(42.85%)
IV	19(50%)	17(54.84%)	2(28.57%)
Baseline MG-ADL score, median (IQR)	7.5(5,10)	8(5,11)	7(4,9)
Baseline MG-QOL 15r score, median (IQR)	22(16,30)	22(16,31)	20(16,25)
Comorbidities, n (%)			
Thymoma	10(26.31%)	9(29.03%)	1(14.28%)
Hypertension	20(52.63%)	16(51.61%)	4(57.14%)
Diabetes mellitus	15(39.47%)	12(38.71%)	3(42.85%)
Thyroid disorders	8(21.05%)	6(19.35%)	2(28.57%)
Peptic ulcer disease	7(18.42%)	6(19.35%)	1(14.28%)
Sleep disorders	9(23.68%)	9(29.03%)	0
Other malignancies	8(21.05%)	7(22.58%)	1(14.28%)
Baseline immunotherapy regimen, n (%)			
Corticosteroid only	5(13.15%)	5(16.12%)	0
Corticosteroid & Tacrolimus	14(36.84%)	12(38.71%)	2(28.57%)
Corticosteroid & Mycophenolate mofetil	7(18.42%)	4(12.90%)	3(42.85%)
Tacrolimus & Mycophenolate mofetil	7(18.42%)	5(16.12%)	2(28.57%)
Tacrolimus only	4(10.52%)	4(12.90%)	0
Mycophenolate mofetil only	1(2.63%)	1(3.22%)	0
Pyridostigmine at baseline, mg,median (IQR)	180(180,180)	180(180,180)	180(180,180)

Data are presented as mean ± SD, median (IQR), or n (%). The primary analysis cohort included patients who continued eculizumab for at least 2 months. The early discontinuation cohort included patients who discontinued eculizumab before 2 months.

Due to the small sample size, baseline characteristics of the early treatment discontinuation cohort were presented descriptively. Compared with the main analysis cohort, patients in this group were older, with divergent distributions of MGFA classification and baseline immunosuppressive regimens, while baseline MG-ADL and MG-QOL 15r scores were generally comparable.

### 3.3 Follow-up duration and retention rate

The shortest follow-up duration of the 31 enrolled patients was 2 months, and the longest was 15 months, with a mean follow-up duration of 10 ± 4.29 months. The 3-, 6-, and 12-month retention rates were 90.32%, 80.65%, and 67.74%, respectively.

### 3.4 Changes in MG-ADL scores

**Figure 1:**
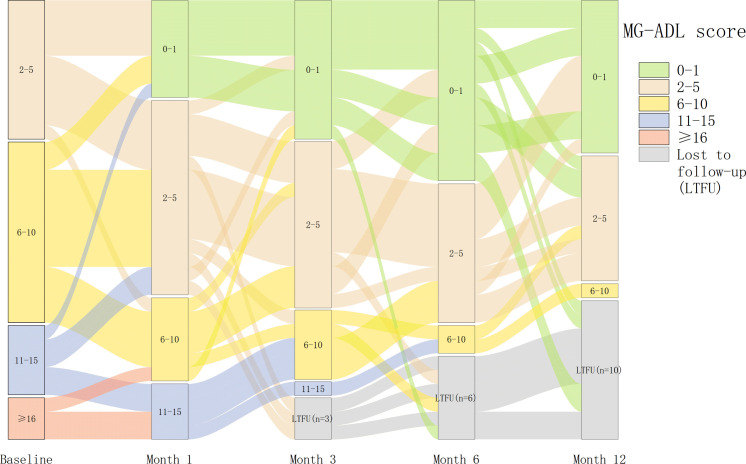
Distribution of MG-ADL scores over time among 31 LOMG patients on eculizumab for at least two months. MG-ADL, myasthenia gravis activities of daily Living.

Alluvial plots were used to visualize the dynamic changes in the distribution of the MG-ADL scores in patients receiving eculizumab treatment (**Figure 1**). At baseline, the patients were mainly concentrated in the higher score ranges (≥6 points). As treatment progressed, the color bands representing the lower score ranges (especially the 0–1 point range corresponding to green) widened, indicating a continuous increase in the proportion of patients achieving mild or asymptomatic status. Meanwhile, the color bands for higher score ranges (≥6 points) narrowed, suggesting a gradual decrease in the proportion of patients with moderate-to-severe symptoms. This plot intuitively reflects the overall trend of patients moving toward a better clinical status.

**Figure 2:**
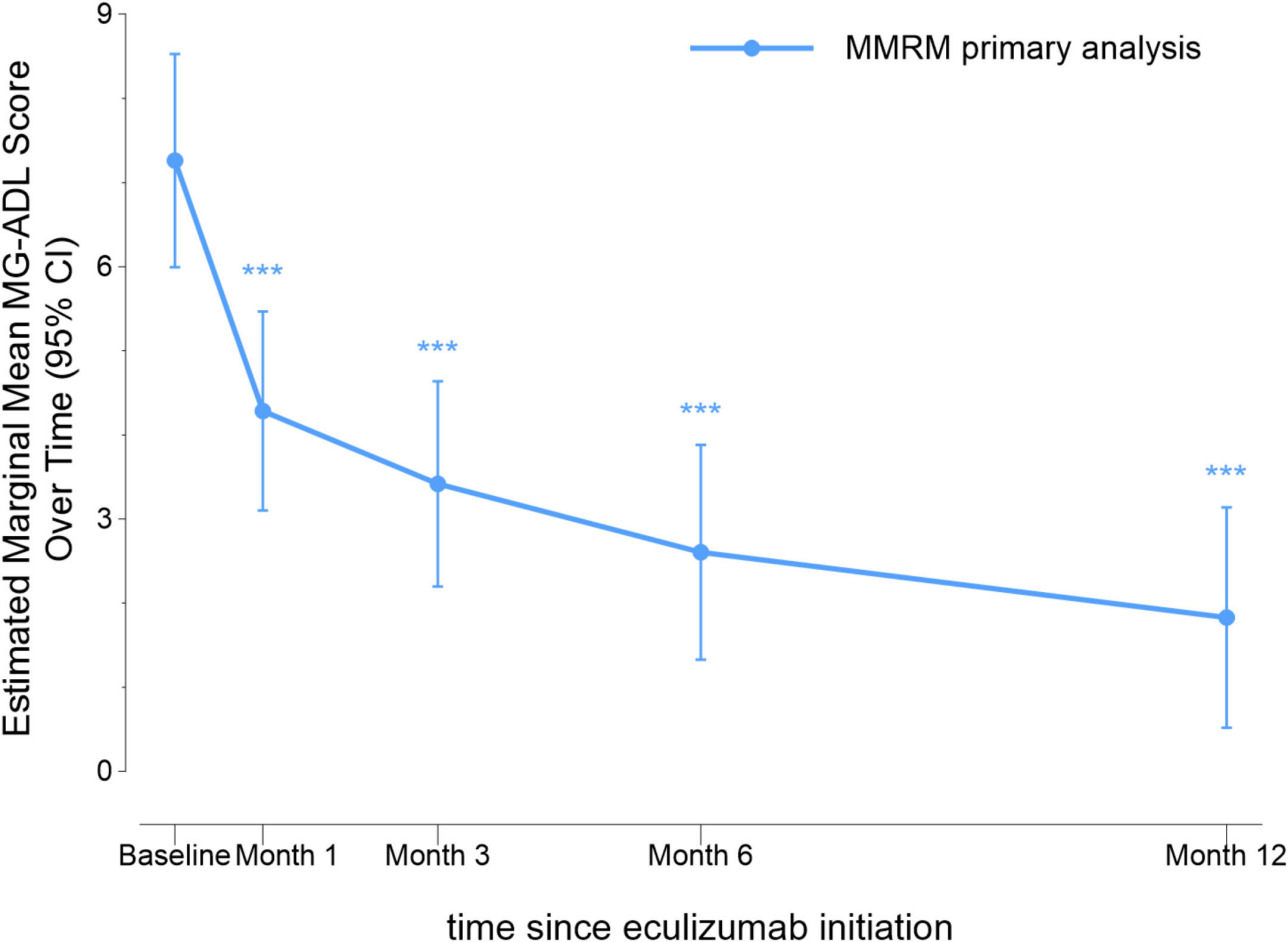
Changes in MG-ADL scores over time among 31 LOMG patients on eculizumab for at least two months. Note: Error bars represent 95% confidence intervals; *** indicates P<0.001 compared with baseline; MG-ADL, myasthenia gravis activities of daily living.

In the MMRM primary analysis, visit had a significant effect on MG-ADL scores (F = 14.453, P < 0.001). The adjusted mean MG-ADL score decreased from 7.261(95% CI, 5.995–8.526) at baseline to 4.285(95% CI, 3.103–5.466), 3.418(95% CI, 2.201–4.635), 2.604(95% CI, 1.329–3.880), 1.831(95% CI, 0.522–3.140) at Month 1, 3, 6, 12, respectively (**Figure 2**; **Supplementary Table 1**). Compared with baseline, MG-ADL scores were significantly reduced at Month 1, 3, 6, and 12, with mean differences of -2.976(95% CI, -4.182 to -1.771), -3.843(95% CI,-5.189 to -2.497), -4.657(95% CI, -6.125 to -3.188), and -5.430(95% CI, -6.995 to -3.864), respectively (all P < 0.001).

A reduction of ≥3 points in the MG-ADL score from baseline was defined as a clinically meaningful improvement (CMI). The cumulative response rate of CMI reached 51.61% at Month 1 after treatment initiation and increased to 78.49% at Month 3, 89.24% at Month 6, and 96.41% at Month 12.

### 3.5 Changes in MG-QOL 15r scores

**Figure 3:**
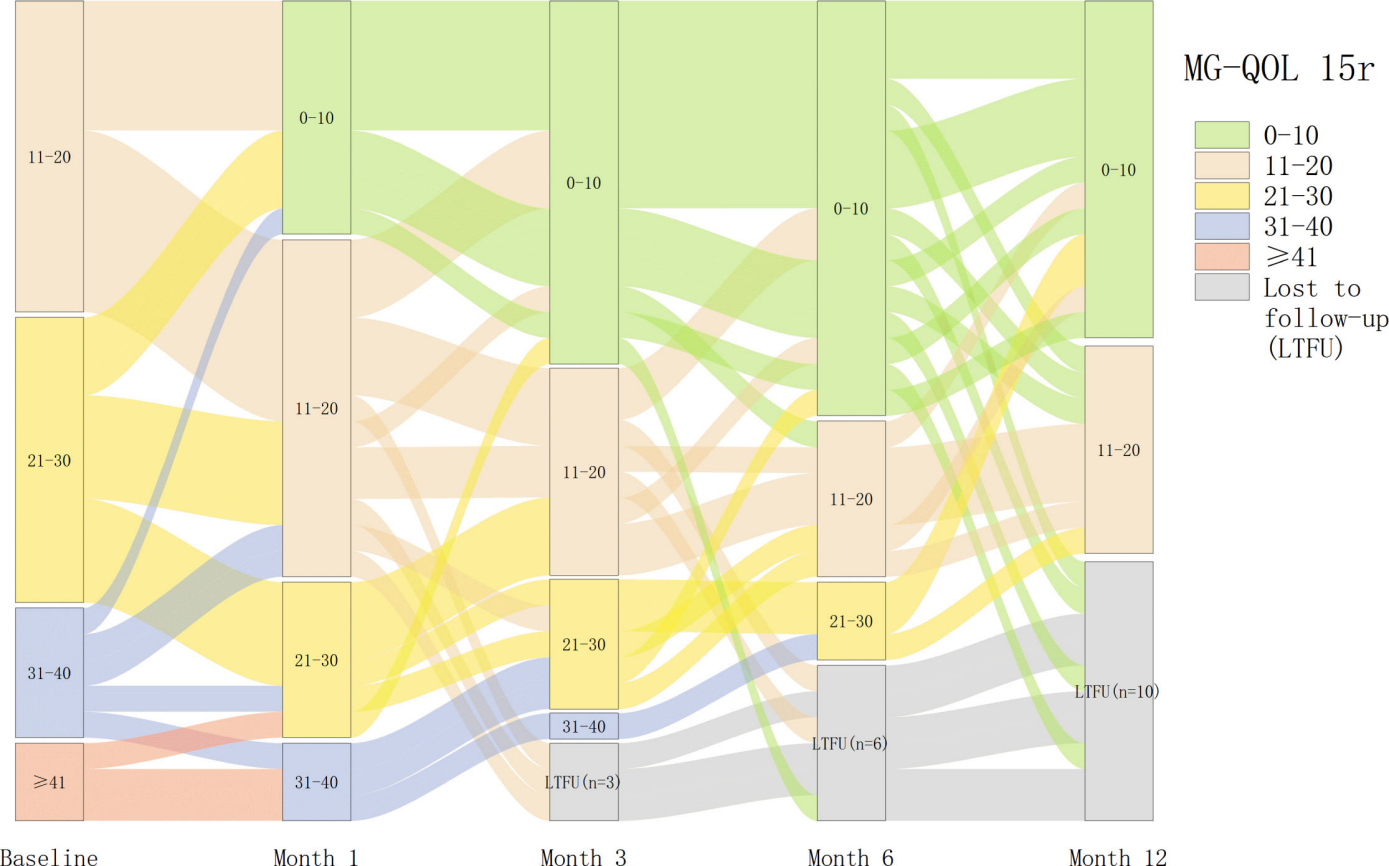
Distribution of MG-QOL 15r scores over time among 31 LOMG patients on eculizumab for at least two months. MG-QOL 15r = 15-item revised myasthenia gravis quality of life questionnaire.

Alluvial plots (**Figure 3**) were used to show the dynamic changes in the distribution of the MG-QOL 15r scores over the 12 months of treatment. With extended treatment time, the patient population showed a shift from higher to lower scores. The color bands representing the lower score ranges (especially the 0–10 point range corresponding to green) widened, indicating continuous improvement in the overall quality of life.

**Figure 4:**
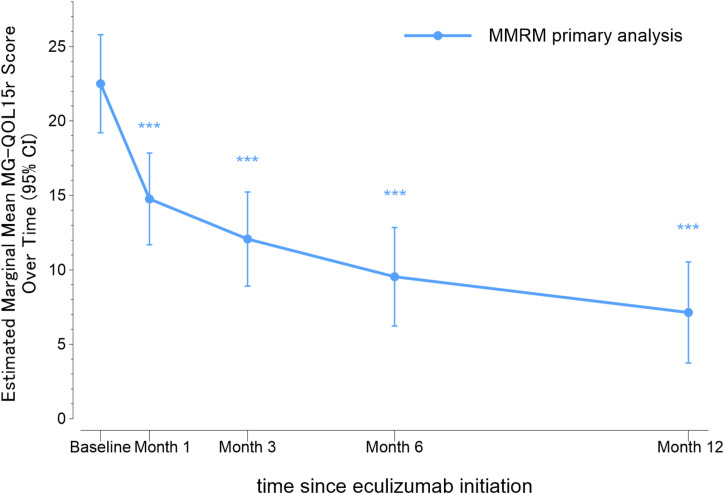
Changes in MG-QOL 15r scores over time among 31 LOMG patients on eculizumab for at least two months. Error bars represent 95% confidence intervals; *** indicates P<0.001 compared with baseline; MG-QOL 15r = 15-item revised myasthenia gravis quality of life questionnaire.

In the MMRM primary analysis, visit had a significant effect on MG-QOL 15r scores (F = 17.624, P<0.001). The adjusted mean MG-QOL 15r score decreased from 22.492 (95% CI, 19.203–25.782) at baseline to 14.768 (95% CI, 11.689–17.848), 12.069 (95% CI, 8.903–15.234), 9.540 (95% CI, 6.229–12.852), and 7.149 (95% CI, 3.761–10.537) at Month 1, 3, 6, and 12, respectively (**Figure 4**; **Supplementary Table 1**). Compared with baseline, MG-QOL 15r scores showed sustained significant reductions at Month 1, 3, 6, and 12, with mean differences of -7.724 (95% CI, -10.733 to -4.715), -10.424 (95% CI, -13.801 to -7.047), -12.952 (95% CI, -16.649 to -9.255), and -15.343 (95% CI, -19.283 to -11.403), respectively (all P<0.001).

### 3.6 Achievement of MSE

**Figure 5:**
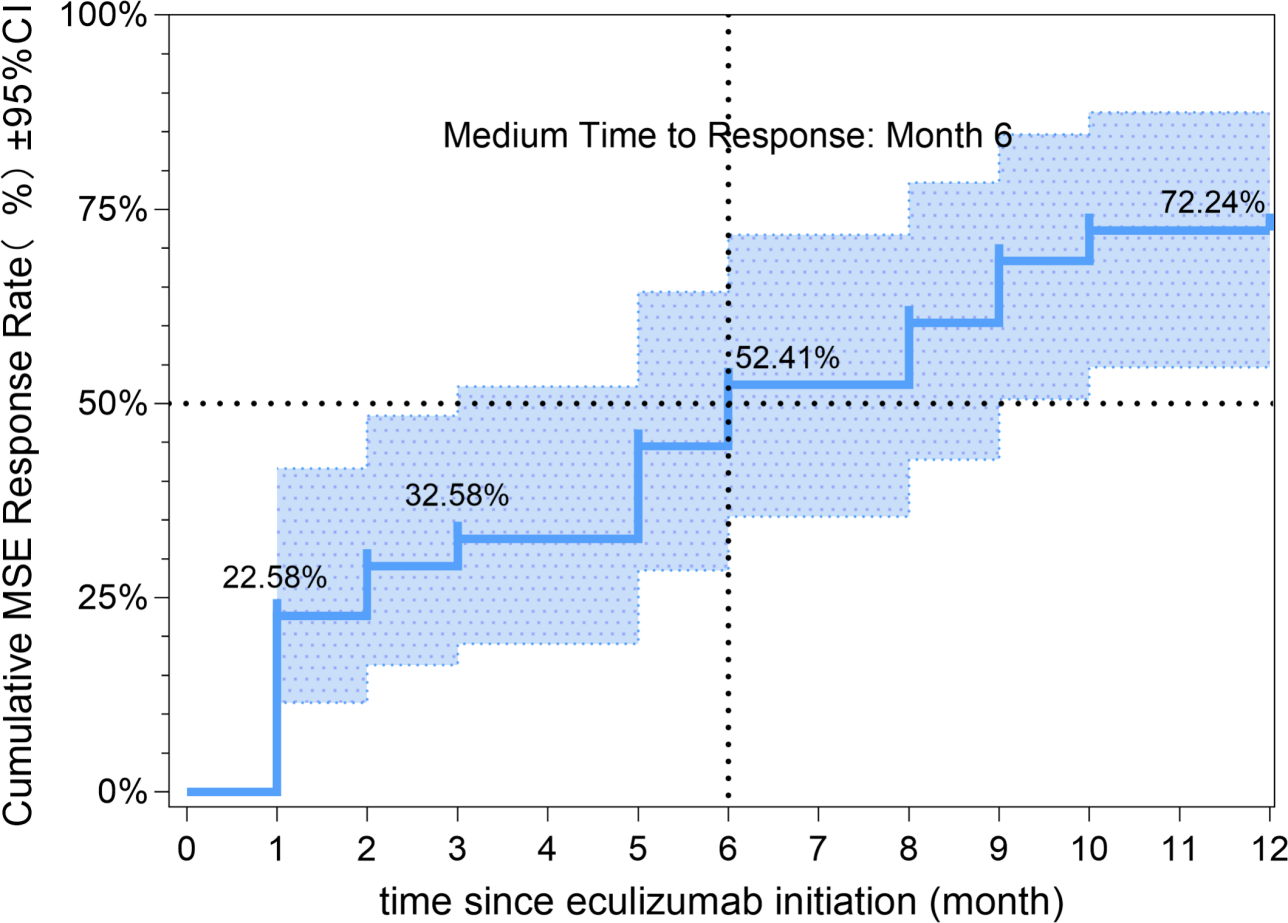
Cumulative MSE response rate over time among 31 LOMG patients on eculizumab for at least two months. Error bars represent 95% confidence intervals; MSE, minimal symptom expression (defined as an MG-ADL score of 0 or 1); MG-ADL, myasthenia gravis activities of daily living; median response time = 6 months.

The status of the MSE response in patients receiving eculizumab treatment was analyzed. Time-to-event analysis was performed using the Kaplan-Meier method to plot the cumulative response rate curve for MSE achievement after the initiation of eculizumab treatment (**Figure 5**). The results showed that the cumulative response rate of MSE increased continuously with increasing treatment duration. The cumulative response rate of MSE reached 22.58% at Month 1 after treatment initiation and increased to 32.58% at Month 3, 52.41% at Month 6, and 72.24% at Month 12. The median time to response MSE was six months.

### 3.7 Longitudinal changes in concomitant background therapies

**Figure 6:**
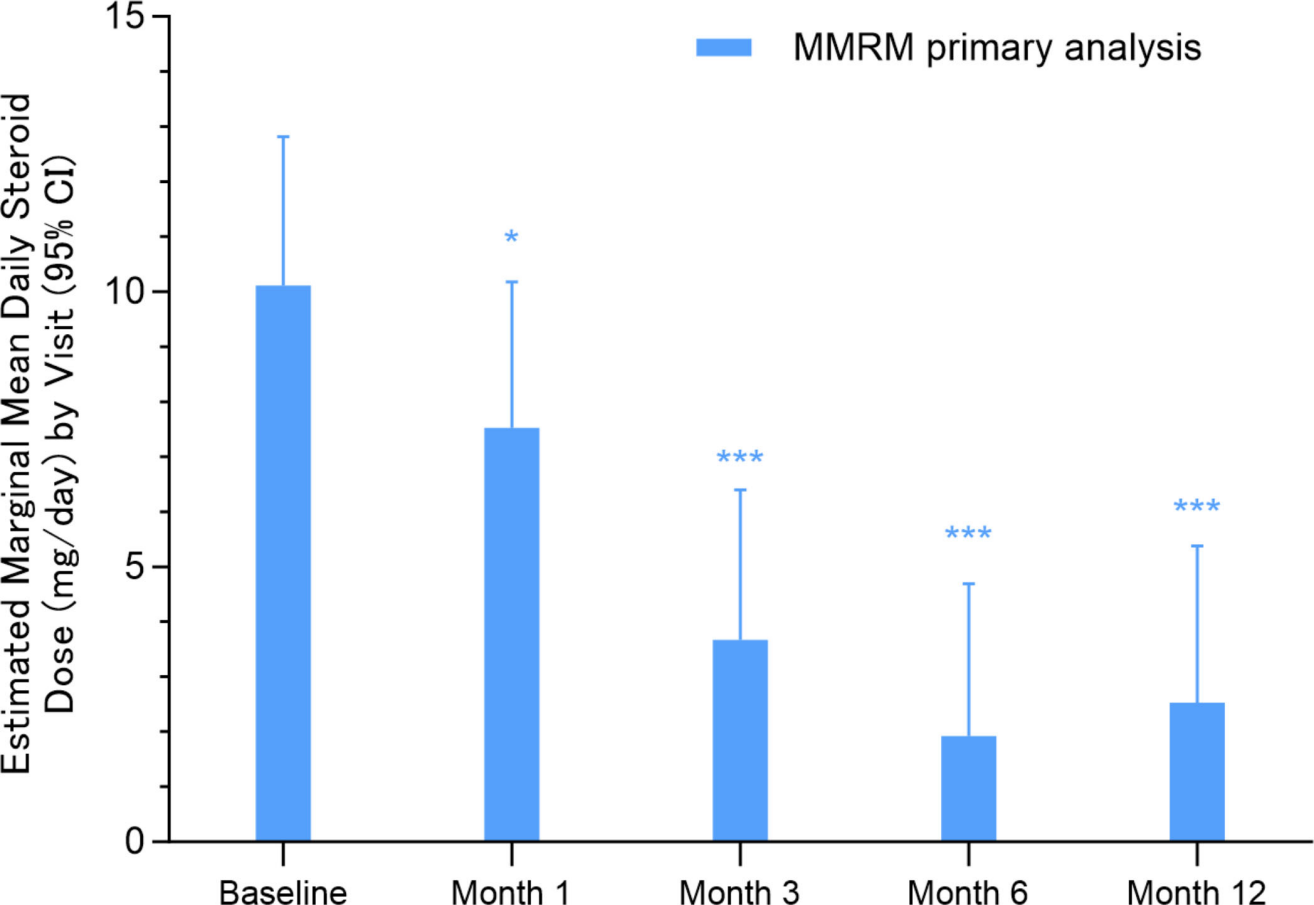
Changes in prednisone dosage over treatment time among 31 LOMG patients on eculizumab for at least two months (mg/day). Error bars represent 95% confidence intervals, *** indicates P<0.001 compared with baseline, * indicates P<0.05.

In the MMRM primary analysis, visit had a significant effect on daily corticosteroid dose (F = 15.713, P<0.001). The adjusted mean corticosteroid dose (mg/day) decreased from 10.112 at baseline to 7.525, 3.675, 1.920, and 2.538 at Month 1, 3, 6, and 12, respectively(**Figure 6**; **Supplementary Table 1**). Compared with baseline, the corticosteroid dose showed sustained significant reductions at Month 1, 3, 6, and 12, with mean differences of -2.587 (95% CI, -4.760 to -0.414), -6.437 (95% CI, -8.703 to -4.172), -8.192 (95% CI, -10.594 to -5.790), and -7.574 (95% CI, -10.238 to -4.910), respectively (all P<0.05).

**Figure 7:**
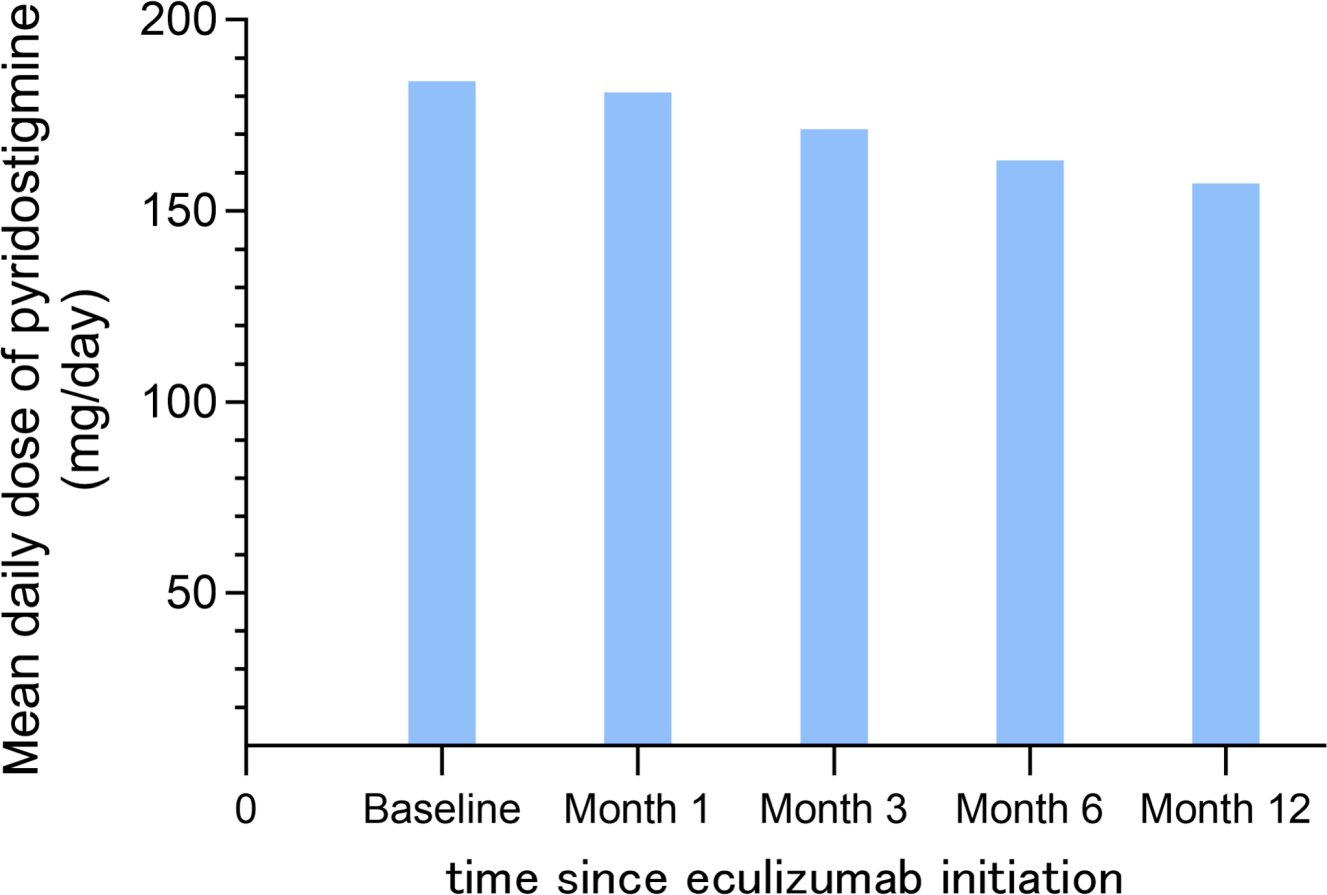
Mean daily dose of pyridostigmine over treatment time among 31 LOMG patients on eculizumab for at least two months (mg/day).

A mild downward trend was observed in the mean daily dose of pyridostigmine from baseline (183.8mg/day) through Month 12(157.1mg/day) during follow-up (**Figure 7**).

**Figure 8:**
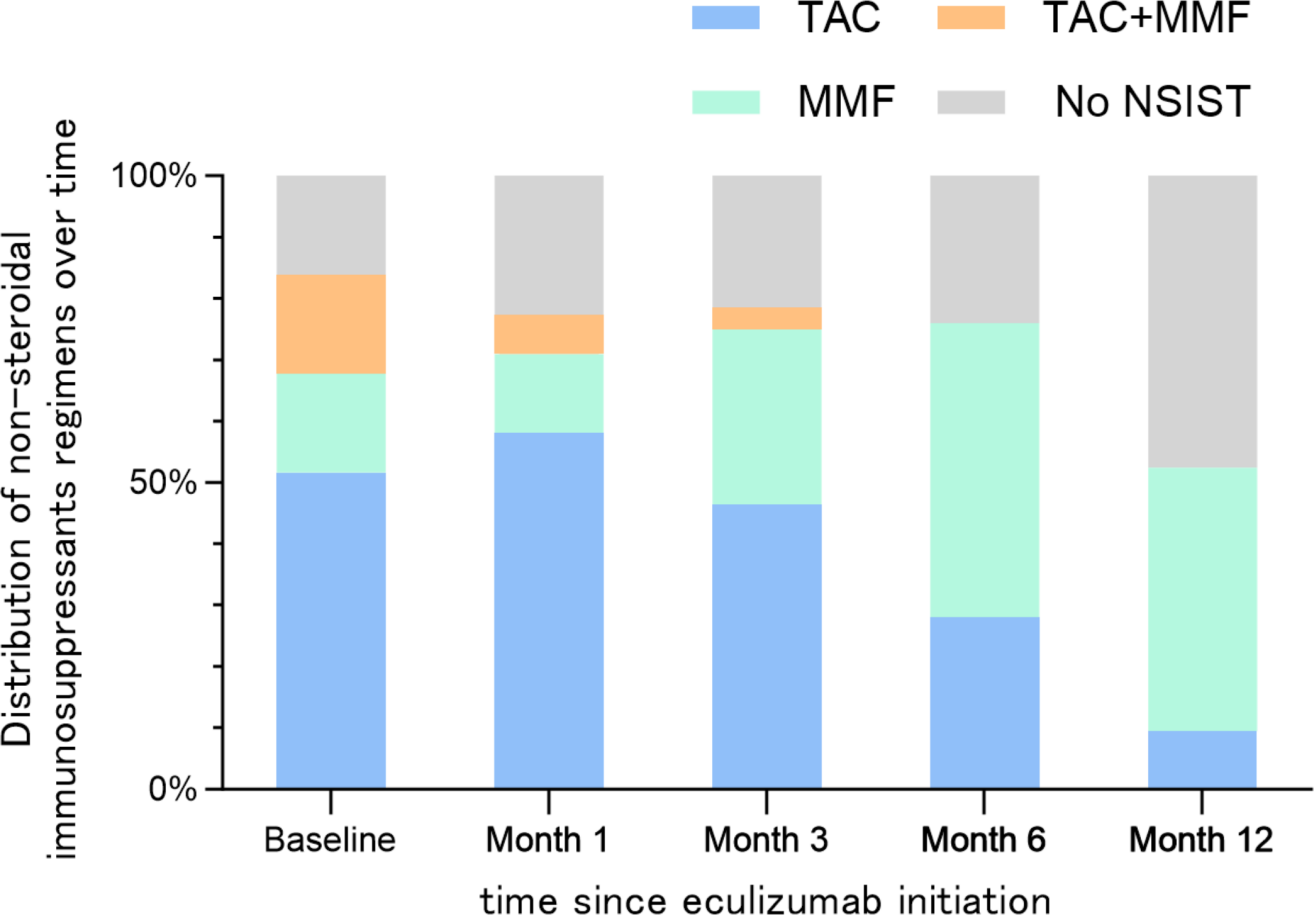
100% stacked bar chart for non-steroidal immunosuppressants use over time among 31 LOMG patients on eculizumab for at least two months. The 100% stacked bars represent the proportion of patients receiving each non−steroidal immunosuppressant regimen at baseline and at Month 1, 3, 6, and 12 after initiation of eculizumab. Regimens include tacrolimus (TAC), mycophenolate mofetil (MMF), combination therapy (TAC + MMF), and no non-steroidal immunosuppressant(no NSIST).

The longitudinal distribution of non-steroidal immunosuppressant (NSIST) regimens during follow-up is presented in **Figure 8**. At baseline, tacrolimus (TAC) monotherapy was the most frequently used regimen (16/31, 51.6%), followed by mycophenolate mofetil (MMF) monotherapy (5/31, 16.1%), combination therapy (TAC + MMF) (5/31, 16.1%), and no non−steroidal immunosuppressant (5/31, 16.1%). Over time, the proportion of patients receiving TAC monotherapy decreased progressively, whereas MMF monotherapy and the proportion without non-steroidal immunosuppressant increased. Combination therapy (TAC + MMF) declined to 0% by Month 6. By Month 12, the proportion of patients receiving TAC monotherapy decreased to 2/21 (9.5%), while MMF monotherapy increased to 9/21 (42.9%). The proportion of patients not receiving any non-steroidal immunosuppressant increased to 10/21 (47.6%). This apparent shift from TAC to MMF was primarily related to temporary supply issues of TAC in the hospital pharmacy during the study period, rather than a lack of clinical efficacy. More importantly, despite this lateral switch in a subset of patients, the proportion of patients not using nonsteroidal immunosuppressants increased from 16.1% (5/31) at baseline to 47.6% (10/21) at Month 12.

### 3.8 Sensitivity analysis

Both sensitivity analyses supported the robustness of the primary findings. In the multiple imputation analysis, MG-ADL, MG-QOL 15r, and corticosteroid dose remained significantly improved from baseline at all post-baseline visits. At Month 12, the estimated changes were -5.152 for MG-ADL, -14.816 for MG-QOL 15r, and -7.641 for corticosteroid dose (Detailed results are shown in **Supplementary Table 2**).

In the conservative BOCF analysis including all 38 treated patients, improvements were attenuated but remained significant. At Month 12, the estimated changes from baseline were -3.948 for MG-ADL, -11.119 for MG-QOL 15r, and -5.751 for corticosteroid dose, all P < 0.001 (Detailed results are shown in **Supplementary Table 3**).

Overall, the multiple imputation analysis yielded results highly consistent with the primary analysis, whereas the conservative BOCF analysis showed smaller effect sizes but did not alter the direction or statistical significance of the findings.

### 3.9 Safety

The duration of safety follow-up among the 38 patients ranged from 0.5 to 15 months, with a mean follow-up of 8.28 months. During the follow-up period, one patient reported a mild adverse event (headache), CTCAE grade 1, which did not require treatment discontinuation. No serious adverse events or severe infections were documented in any patient.

## 4 Discussion

Eculizumab precisely inhibits complement C5 and blocks the formation of the MAC and its damage to the NMJ, thus enabling rapid control of disease progression (20, 21). The REGAIN phase III clinical trial and other studies confirmed that eculizumab has the characteristics of rapid onset, long-term maintenance of efficacy, and favorable safety in refractory AChR-Ab-positive generalized MG (18, 19, 22). However, the mean age of onset in the REGAIN study population was 38.0 years, the mean disease duration was 9.9 years, and the majority of patients had mild to moderate cases (MGFA type II/III, 88%) (18), which has limited representativeness for elderly patients. Patients with refractory LOMG have multiple comorbidities and unmet treatment needs (5). In this study, the mean age at the onset of MG was 62.87 years, the median disease duration was 27 months, and 54.84% were MGFA type IV severe cases, indicating that LOMG is associated with faster disease progression and higher disease severity. Therefore, the choice of treatment for LOMG is important. This study evaluated the clinical outcomes of eculizumab in a refractory LOMG population, its safety profile, and impact on quality of life. The results showed that eculizumab treatment was associated with rapid symptom relief in the short term and sustained long-term clinical benefits during follow-up, as well as improvements in activities of daily living and HRQOL. These findings provide real-world data on the potential benefits of eculizumab in this specific subgroup, although confirmation in controlled studies is warranted.

The rapid onset of action of eculizumab is crucial for elderly patients with MG with low physiological reserves who cannot tolerate disease recurrence and treatment-related impacts. During the progression of LOMG, patients are highly prone to falling into a vicious circle of “worsening myasthenia - prolonged bed rest - secondary infection or thrombosis - myasthenic crisis.” This study showed that the MG-ADL score was reduced by 2.976 points (P < 0.001) 1 month after eculizumab treatment and the reduction reached 3.843 points (P < 0.001) at Month 3, which is comparable to the results of the REGAIN study (a reduction of approximately 3.7 points, p=0.0183) (18). Meanwhile, Kaplan-Meier analysis showed that 22.58% of patients achieved MSE and 51.61% achieved CMI (a reduction of ≥3 points in the MG-ADL score) at 1 month after treatment, and these proportions increased to 32.58% and 78.49% at Month 3, respectively. This indicates that a rapid response to eculizumab correlates with a reduction in disease activity and improvements in activities of daily living in the short term.

Consistent with the results of the REGAIN trial, our study observed that the patients’ clinical symptoms continued to improve following eculizumab treatment, and these clinical benefits were stably maintained in the long term. After a reduction of 2.976 points in the MG-ADL score from baseline at Month 1 of treatment, the reduction further expanded to 3.843 points, 4.657 points, and 5.430 points at Month 3, 6, and 12. The cumulative response rate for achieving MSE also increased gradually over time, rising from 22.58% at Month 1 to 32.58% at Month 3, 52.41% at Month 6, and 72.24% at Month 12. These findings suggest a potential association between longer treatment duration and improved clinical outcomes in our cohort.

Most existing studies consider muscle strength scores or symptom control as the primary endpoints; however, the core demand of elderly patients with MG is to restore their independent living ability and reduce their dependence on others. Therefore, for this population, the evaluation of therapeutic efficacy should not be limited to symptom improvement but should focus on the comprehensive quality of life in multiple dimensions, including psychological, social, and functional status. In this study, after 12 months of eculizumab treatment, the MG-QOL 15r and MG-ADL scores of patients reduced by 15.343 points and 5.430 points from baseline, respectively, indicating meaningful improvements in patients’ self-care ability, psychological state, and social functioning. In our cohort of refractory LOMG patients, eculizumab treatment was associated with favorable improvements in quality of life. These preliminary findings suggest that in this specific, difficult-to-treat population, the therapeutic goal may extend beyond mere symptomatic relief to include quality-of-life enhancement.

Eculizumab is known to increase susceptibility to meningococcal infection due to complement C5 inhibition. In our cohort, all patients received meningococcal vaccination prior to treatment initiation or received prophylactic antibiotics during treatment, and no cases of meningococcal infection or other severe opportunistic infections were documented during follow-up to date. Although these findings are reassuring, they should be interpreted cautiously. The relatively small sample size, limited follow-up duration, and retrospective data collection may potentially lead to an underestimation of the true incidence of adverse events, particularly mild or subclinical infections. In addition, infection monitoring was based on routine clinical practice rather than a predefined study-specific surveillance protocol. Therefore, larger prospective studies with standardized safety monitoring are warranted to more comprehensively characterize the safety profile of eculizumab in this population.

Furthermore, eculizumab treatment was associated with a decreased requirement for concomitant immunosuppressants. The daily dosage of prednisone was reduced by 2.587 mg/day and 7.574 mg/day from baseline at Month 1 and 12 after treatment, respectively. Concomitant use of nonsteroidal immunosuppressants decreased from 83.9% at baseline to 52.4% at Month 12. Taken together, these findings suggest a potential reduction in overall immunosuppressive burden in part of the cohort. In elderly patients with MG, such reductions may be clinically relevant, as lower cumulative exposure to glucocorticoids and other immunosuppressants could be associated with a decreased risk of treatment-related adverse effects, including hypertension, hyperglycemia, and osteoporosis. In addition, complement C5 inhibition does not directly suppress T-cell or B-cell function (20, 21),representing a mechanistically distinct approach compared with broad immunosuppressive therapies.

An important observation in this real-world study is the dynamic change in concomitant background therapies. While eculizumab treatment was associated with reduced glucocorticoid requirements, changes in non-steroidal immunosuppressants warrant cautious interpretation. Specifically, we observed a decrease in TAC use alongside an increase in MMF. In our clinical setting, this lateral drug switch was primarily necessitated by a temporary institutional supply shortage of tacrolimus rather than by poor clinical efficacy or adverse events. Although some patients underwent a mandatory switch to MMF, a proportion of patients were able to discontinue non-steroidal immunosuppressants during follow-up. Taken together, these findings suggest a possible reduction in overall immunosuppressive exposure in part of the cohort; however, given the dynamic adjustment of background therapies in this observational setting, these changes should be interpreted cautiously.

Given that concomitant therapies are a major source of confounding, it can be challenging to attribute the observed clinical improvements solely to eculizumab. To rigorously address this and account for the fluctuations in background medications, we included follow-up daily doses of glucocorticoids and pyridostigmine, as well as non-steroidal immunosuppressant use, as time-varying covariates in our MMRM model. The results from this adjusted model demonstrated statistically significant improvements in clinical outcomes over the follow-up period. These findings suggest that the positive association between eculizumab treatment and clinical improvements appears to be robust after adjusting for changes in concomitant therapies. Nevertheless, due to the retrospective design and the absence of a control group, causal inferences cannot be established.

This single-center retrospective study had a relatively small sample size and incomplete follow-up. A notable limitation of this study is the potential for selection bias and attrition bias in our analysis of clinical outcomes. The relatively low follow-up retention at Month 12 may have introduced attrition bias due to missing follow-up data. To assess the influence of missing follow-up data, we performed a multiple imputation sensitivity analysis with 50 imputed datasets followed by the same MMRM models in the primary efficacy population. In addition, the exclusion of seven patients who discontinued eculizumab before 2 months may have introduced selection bias and potentially overestimated treatment benefit, particularly if early discontinuation was related to poor efficacy or other non-random reasons. To address this concern, we performed a conservative all-treated BOCF sensitivity analysis including all 38 patients who received eculizumab, assuming no post-baseline clinical improvement or steroid-sparing effect among early discontinuers. Both sensitivity analyses produced results consistent with the primary analysis. The multiple imputation analysis showed findings highly similar to the primary MMRM results, suggesting that missing follow-up data did not substantially alter the conclusions. Although the conservative BOCF analysis attenuated the magnitude of improvement, significant improvements in MG-ADL, MG-QOL 15r, and corticosteroid dose were still observed. These findings support the robustness of the observed clinical benefits of eculizumab. Nevertheless, given the observational design, small sample size, loss to follow-up, and non-random treatment discontinuation, residual bias cannot be fully excluded, and the findings should be interpreted cautiously. Statistically, LSD test was applied for repeated pairwise comparisons against baseline, without multiple testing correction. Considering the exploratory attribute and limited sample size, strict correction would impair statistical power and fail to reflect actual clinical changing trends. The obtained significant differences are preliminary findings and need verification in subsequent large-scale studies. Moreover, the number of patients using non-steroidal immunosuppressants was small, which limited the statistical power of the analysis, and the relevant results were only presented descriptively. The findings of this study are merely preliminary real-world data from a restricted cohort. All results should be interpreted cautiously considering study limitations. Large multicenter prospective controlled studies with extended follow-up are required to further validate the long-term efficacy and safety of eculizumab in patients with LOMG. It is recommended that patient quality of life indicators be included as the primary study endpoints. Individualized treatment strategies and optimal intervention times should be explored in combination with complement-related biomarkers and patient characteristics, and cost-effectiveness analyses should be conducted to provide sufficient evidence for the clinical promotion and rational application of this targeted therapy.

## 5 Conclusion

This study provides preliminary real-world evidence on eculizumab treatment in patients with refractory LOMG, complex comorbidities, and poor tolerance to conventional therapy. Eculizumab treatment was associated with a rapid clinical improvement, sustained improvement in quality of life and reduced corticosteroid dose. However, these preliminary real-world findings require further validation in larger prospective controlled studies.

## Data Availability

The raw data supporting the conclusions of this article will be made available by the authors, without undue reservation.
