# Supplementary material for: Clinical outcomes and quality of life associated with eculizumab in patients with late-onset myasthenia gravis
Source: Front Immunol. 2026 Jun 12;17:1834680. doi: 10.3389/fimmu.2026.1834680 (PMC13303812; doi:10.3389/fimmu.2026.1834680)
Supplement: Supplementary file 1 [file Table1.docx]

**Supplementary Table. Primary and sensitivity analyses of longitudinal outcomes after eculizumab treatment**

**Table S1. Primary MMRM analysis.**

| **Outcome** | **Visit** | **EMM** | **SE** | **95% CI** | **Change from baseline** | **SE of change** | **95% CI of change** | **P value** |
| --- | --- | --- | --- | --- | --- | --- | --- | --- |
| MG-ADL | Baseline | 7.261 | 0.636 | 5.995 to 8.526 | Reference | — | — | — |
|  | Month 1 | 4.285 | 0.593 | 3.103 to 5.466 | -2.976 | 0.607 | -4.182 to -1.771 | <0.001 |
|  | Month 3 | 3.418 | 0.612 | 2.201 to 4.635 | -3.843 | 0.679 | -5.189 to -2.497 | <0.001 |
|  | Month 6 | 2.604 | 0.642 | 1.329 to 3.880 | -4.657 | 0.742 | -6.125 to -3.188 | <0.001 |
|  | Month 12 | 1.831 | 0.659 | 0.522 to 3.140 | -5.430 | 0.791 | -6.995 to -3.864 | <0.001 |
| MG-QOL | Baseline | 22.492 | 1.651 | 19.203 to 25.782 | Reference | — | — | — |
|  | Month 1 | 14.768 | 1.544 | 11.689 to 17.848 | -7.724 | 1.516 | -10.733 to -4.715 | <0.001 |
|  | Month 3 | 12.069 | 1.588 | 8.903 to 15.234 | -10.424 | 1.704 | -13.801 to -7.047 | <0.001 |
|  | Month 6 | 9.540 | 1.663 | 6.229 to 12.852 | -12.952 | 1.867 | -16.649 to -9.255 | <0.001 |
|  | Month 12 | 7.149 | 1.704 | 3.761 to 10.537 | -15.343 | 1.989 | -19.283 to -11.403 | <0.001 |
| Corticosteroid dose | Baseline | 10.112 | 1.351 | 7.410 to 12.815 | Reference | — | — | — |
|  | Month 1 | 7.525 | 1.324 | 4.872 to 10.178 | -2.587 | 1.095 | -4.760 to -0.414 | 0.020 |
|  | Month 3 | 3.675 | 1.361 | 0.954 to 6.396 | -6.437 | 1.142 | -8.703 to -4.172 | <0.001 |
|  | Month 6 | 1.920 | 1.389 | -0.856 to 4.696 | -8.192 | 1.211 | -10.594 to -5.790 | <0.001 |
|  | Month 12 | 2.538 | 1.424 | -0.301 to 5.378 | -7.574 | 1.343 | -10.238 to -4.910 | <0.001 |

*Notes: BOCF, baseline observation carried forward. CI, confidence interval; EMM, estimated marginal mean; SE, standard error; MMRM, mixed model for repeated measures; MG-ADL, Myasthenia Gravis Activities of Daily Living; MG-QOL, Myasthenia Gravis Quality of Life. Primary analysis included patients who remained on eculizumab treatment for at least 2 months. Dash indicates not applicable.*

**Table S2. Multiple imputation sensitivity analysis with 50 imputed datasets followed by MMRM.**

| **Outcome** | **Visit** | **EMM** | **SE** | **95% CI** | **Change from baseline** | **SE of change** | **P value** |
| --- | --- | --- | --- | --- | --- | --- | --- |
| MG-ADL | Baseline | 7.254 | 0.659 | 5.961 to 8.546 | Reference | — | — |
|  | Month 1 | 4.261 | 0.612 | 3.062 to 5.461 | -2.992 | 0.615 | <0.001 |
|  | Month 3 | 3.420 | 0.632 | 2.180 to 4.659 | -3.834 | 0.693 | <0.001 |
|  | Month 6 | 2.745 | 0.656 | 1.458 to 4.032 | -4.509 | 0.756 | <0.001 |
|  | Month 12 | 2.102 | 0.649 | 0.828 to 3.376 | -5.152 | 0.783 | <0.001 |
| MG-QOL | Baseline | 22.382 | 1.706 | 19.039 to 25.725 | Reference | — | — |
|  | Month 1 | 14.639 | 1.585 | 11.532 to 17.745 | -7.743 | 1.529 | <0.001 |
|  | Month 3 | 12.016 | 1.618 | 8.843 to 15.189 | -10.366 | 1.722 | <0.001 |
|  | Month 6 | 9.783 | 1.681 | 6.486 to 13.079 | -12.599 | 1.883 | <0.001 |
|  | Month 12 | 7.566 | 1.664 | 4.301 to 10.831 | -14.816 | 1.529 | <0.001 |
| Corticosteroid dose | Baseline | 10.007 | 1.400 | 7.264 to 12.751 | Reference | — | — |
|  | Month 1 | 7.421 | 1.372 | 4.732 to 10.109 | -2.587 | 1.102 | 0.019 |
|  | Month 3 | 3.487 | 1.409 | 0.725 to 6.250 | -6.520 | 1.157 | <0.001 |
|  | Month 6 | 1.519 | 1.473 | -1.371 to 4.409 | -8.488 | 1.263 | <0.001 |
|  | Month 12 | 2.367 | 1.448 | -0.474 to 5.207 | -7.641 | 1.376 | <0.001 |

*Notes: BOCF, baseline observation carried forward. CI, confidence interval; EMM, estimated marginal mean; SE, standard error; MMRM, mixed model for repeated measures; MG-ADL, Myasthenia Gravis Activities of Daily Living; MG-QOL, Myasthenia Gravis Quality of Life. Multiple imputation sensitivity analysis used 50 imputed datasets followed by repeated fitting of the same MMRM model; pooled estimates are reported. Dash indicates not applicable.*

**Table S3. Conservative BOCF sensitivity analysis in the all-treated population.**

| **Outcome** | **Visit** | **EMM** | **SE** | **95% CI** | **Change from baseline** | **SE of change** | **95% CI of change** | **P value** |
| --- | --- | --- | --- | --- | --- | --- | --- | --- |
| MG-ADL | Baseline | 7.483 | 0.640 | 6.208 to 8.757 | Reference | — | — | — |
|  | Month 1 | 5.149 | 0.613 | 3.927 to 6.372 | -2.333 | 0.515 | -3.352 to -1.314 | <0.001 |
|  | Month 3 | 4.607 | 0.621 | 3.369 to 5.844 | -2.876 | 0.556 | -3.976 to -1.777 | <0.001 |
|  | Month 6 | 4.066 | 0.630 | 2.813 to 5.320 | -3.416 | 0.593 | -4.588 to -2.245 | <0.001 |
|  | Month 12 | 3.535 | 0.621 | 2.299 to 4.771 | -3.948 | 0.623 | -5.180 to -2.716 | <0.001 |
| MG-QOL | Baseline | 22.600 | 1.683 | 19.244 to 25.956 | Reference | — | — | — |
|  | Month 1 | 16.568 | 1.615 | 13.345 to 19.792 | -6.031 | 1.307 | -8.619 to -3.443 | <0.001 |
|  | Month 3 | 14.819 | 1.634 | 11.559 to 18.078 | -7.781 | 1.417 | -10.582 to -4.980 | <0.001 |
|  | Month 6 | 13.106 | 1.655 | 9.807 to 16.405 | -9.494 | 1.513 | -12.484 to -6.504 | <0.001 |
|  | Month 12 | 11.481 | 1.632 | 8.228 to 14.735 | -11.119 | 1.590 | -14.261 to -7.976 | <0.001 |
| Corticosteroid dose | Baseline | 11.381 | 1.727 | 7.917 to 14.846 | Reference | — | — | — |
|  | Month 1 | 9.312 | 1.707 | 5.885 to 12.739 | -2.069 | 0.938 | -3.926 to -0.213 | 0.029 |
|  | Month 3 | 6.208 | 1.723 | 2.750 to 9.666 | -5.173 | 0.972 | -7.096 to -3.250 | <0.001 |
|  | Month 6 | 4.917 | 1.729 | 1.449 to 8.384 | -6.465 | 1.019 | -8.481 to -4.448 | <0.001 |
|  | Month 12 | 5.630 | 1.717 | 2.185 to 9.075 | -5.751 | 1.103 | -7.932 to -3.570 | <0.001 |

*Notes: BOCF, baseline observation carried forward. CI, confidence interval; EMM, estimated marginal mean; SE, standard error; MMRM, mixed model for repeated measures; MG-ADL, Myasthenia Gravis Activities of Daily Living; MG-QOL, Myasthenia Gravis Quality of Life. In the conservative BOCF sensitivity analysis, all 38 treated patients were included. For the seven patients who discontinued eculizumab before 2 months, all post-baseline MG-ADL, MG-QOL, and corticosteroid dose values at months 1, 3, 6, and 12 were imputed using their respective baseline values. For the remaining 31 patients, observed data were used and missing follow-up values were left missing. Dash indicates not applicable.*
